# Association between HLA alleles and sub-phenotype of childhood steroid-sensitive nephrotic syndrome

**DOI:** 10.1007/s12519-021-00489-y

**Published:** 2022-01-01

**Authors:** Hao Lee, Li Wang, Fen-Fen Ni, Xue-Ying Yang, Shi-Pin Feng, Xiao-Jie Gao, Huan Chi, Ye-Tao Luo, Xue-Lan Chen, Bao-Hui Yang, Jun-Li Wan, Jia Jiao, Dao-Qi Wu, Gao-Fu Zhang, Mo Wang, Hai-Ping Yang, Han Chan, Qiu Li

**Affiliations:** 1 Pediatric Research Institute, Department of Nephrology, Ministry of Education Key Laboratory of Child Development and Disorders, National Clinical Research Center for Child Health and Disorders, China International Science and Technology Cooperation base of Child development and Critical Disorders, Chongqing Key Laboratory of Pediatrics, Children’s Hospital of Chongqing Medical University, Chongqing, China; 2 Department of Nephrology, Chengdu Women and Children Central Hospital, Chengdu, 610041 China; 3 Department of Nephrology, Shenzhen Children’s Hospital, Shenzhen, China; 4 Department of Statistics, Children’s Hospital of Chongqing Medical University, Chongqing, China

**Keywords:** Human leukocyte antigen, Immunoregulation, Mediating effect, Steroid-sensitive nephrotic syndrome, Sub-phenotypes

## Abstract

**Background:**

Few studies have addressed the effects of human leukocyte antigen (HLA) alleles on different clinical sub-phenotypes in childhood steroid-sensitive nephrotic syndrome (SSNS), including SSNS without recurrence (SSNSWR) and steroid-dependent nephrotic syndrome/frequently relapse nephrotic syndrome (SDNS/FRNS). In this study, we investigated the relationship between HLA system and children with SSNSWR and SDNS/FRNS and clarified the value of HLA allele detection for precise typing of childhood SSNS.

**Methods:**

A total of 241 Chinese Han individuals with SSNS were genotyped using GenCap-WES Capture Kit, and four-digit resolution HLA alleles were imputed from available Genome Wide Association data. The distribution and carrying frequency of HLA alleles in SSNSWR and SDNS/FRNS were investigated. Additionally, logistic regression and mediating effects were used to examine the relationship between risk factors for disease process and HLA system.

**Results:**

Compared with SSNSWR, significantly decreased serum levels of complement 3 (C3) and complement 4 (C4) at onset were detected in SDNS/FRNS (C3, *P* < 0.001; C4, *P* = 0.018). The average time to remission after sufficient initial steroid treatment in SDNS/FRNS was significantly longer than that in SSNSWR (*P* = 0.0001). Low level of C4 was further identified as an independent risk factor for SDNS/FRNS (*P* = 0.008, odds ratio = 0.174, 95% confidence interval 0.048–0.630). The HLA-A*11:01 allele was independently associated with SSNSWR and SDNS/FRNS (*P* = 0.0012 and *P* = 0.0006, respectively). No significant HLA alleles were detected between SSNSWR and SDNS/FRNS. In addition, a mediating effect among HLA-I alleles (HLA-B*15:11, HLA-B*44:03 and HLA-C*07:06), C4 level and SDNS/FRNS was identified.

**Conclusions:**

HLA-I alleles provide novel genetic markers for SSNSWR and SDNS/FRNS. HLA-I antigens may be involved in steroid dependent or frequent relapse in children with SSNS as mediators of immunoregulation.

**Supplementary Information:**

The online version contains supplementary material available at 10.1007/s12519-021-00489-y.

## Introduction

Idiopathic renal syndrome (INS) is the most common cause of chronic glomerular disease in children. The occurrence and development of INS could be affected by infection, abnormal lipid metabolism, immune disorders, circulating factors and the use of nonsteroidal anti-inflammatory drugs. Childhood INS is with a prevalence of approximately 16 cases per 100,000 children. Nevertheless, existing theories cannot fully account for the pathogenesis and clinical characteristics of this syndrome [1–4]. Indeed, steroid therapy is the most common treatment for INS and approximately 80–90% of childhood INS patients achieve remission with such therapy. However, 20–30% of the cases progress to infrequent relapses and without recurrence (SSNSWR), who can be managed by steroids alone. Noteworthily, approximately 50% of children with initial SSNS exhibit decreased hormone sensitivity and increased likelihood of relapse during infection, steroid tapering or soon after discontinuation. These patients require second-line steroid-sparing therapy and can progress to a frequently relapse (i.e., frequently relapse nephrotic syndrome, FRNS) or steroid-dependent course (i.e., steroid-dependent nephrotic syndrome, SDNS) [5, 6]. Moreover, a small proportion of children with FRNS or SDNS still undergo relapses or exhibit active disease in adulthood, accompanied by long-term adverse effects including renal interstitial damage, hypertension, diabetes, dyslipidemia, cataracts, osteoporosis, fracture, obesity, growth disorders, infertility and tumors.

In recent years, some researches have attached great importance to genetic mechanisms and revealed genetic mutations existing in 20–30% of patients with SRNS. To date, the causative mutations in more than 70 different genes have been identified to participate in renal dysfunction, including slit diaphragms, actin cytoskeletons, mitochondrial proteins involved in the biosynthesis of coenzyme Q10, and basement membranes in glomeruli [7–9]. However, the etiology of most SSNS cases remains unclear, of which immune disorders are considered to be a key factor affecting the occurrence and development [10–12]. It is worth emphasizing that human leukocyte antigen (HLA) allele polymorphisms are associated with a variety of kidney diseases [13–16]. Strong associations among HLA alleles, especially HLA-II alleles, and their susceptibility to SSNS have been reported to be correlated with HLA-DQA1 and HLA-DQB1.

Highly variable HLA, the expression products of human major histocompatibility complex (MHC) molecules, present antigen peptides to T cells, thus triggering activation of immune complexes and regulating immune homeostasis. Intense investigations have identified HLA genes as inherited risk factors for conditions linked with autoimmunity. Previous findings have confirmed that the polymorphisms of HLA alleles could affect constant region peptide-binding grooves, which determines the antigen peptide repertoire [17]. During the immune response, HLA allele polymorphisms could affect the generation of peptides, antigen binding and antigen presentation, thereby affecting disease progression in SSNS patients. Previous studies on the association between HLA alleles and characteristics of SSNS have mostly focused on the identification of disease-related loci, and the populations included were small and mostly included patients with a single phenotype. Furthermore, a simple comparison of HLA type differences between SSNS patients and healthy controls may exclude the effects of indicators related to disease development and prognosis. Previous studies have highlighted the strong association between HLA and its susceptibility to SSNS [18–23]. However, ethnic and regional differences in HLA allele polymorphisms may lead to genetic heterogeneity [24]. Analysis of the correlation between HLA allele polymorphisms and characteristics of SSNS requires detailed differentiation. Our clinical diagnosis and treatment of SSNS showed significant difference regarding the clinical characteristics between SSNSWR and SDNS/FRNS children, suggesting various genetic characteristics in two groups. Previous studies mostly used polymerase chain reaction (PCR) to type the target alleles, from the earliest PCR-restriction fragment length polymorphism to PCR-sequence specific oligonucleotides and then to the most commonly used PCR-sequence specific primer. These methods have the advantages of simplicity, sensitivity, low sample demand and low cost. However, the typing process has low throughput, is not easy to automate, is prone to errors, and cannot detect new genes and loci. Therefore, limitations remain in the study of diseases and HLA alleles. Compared with traditional HLA analysis methods, next generation sequencing (NGS) has higher resolution and larger sample throughput. Moreover, genetic heterogeneity exists in children with SSNS of different races, regions and clinical phenotypes [25–27]. Therefore, the associations among HLA alleles and different clinical phenotypes of SSNS (SSNSWR or SDNS/FRNS) still remain to be further studied. In this study, we used NGS to analyze the association between HLA alleles and SSNSWR and SDNS/FRNS characteristics in Chinese Han children, to explore the differences of HLA allele distribution and carrier frequency among groups, and to explore the potential impact of HLA allele polymorphism on clinical characteristics. Treatment response and prognosis are helpful to drug screening, clinical classification, and individualized treatment.

## Methods

### Study participants

All samples in this study were collected from children diagnosed with SSNS from Children's Hospital of Chongqing Medical University in China from January 1, 2010 to December 30, 2016. All children in the study met the inclusion and exclusion criteria, and the clinical groups were confirmed by specialized physicians (Supplementary Table 1) [28], SSNSWR means the urine protein turned negative after 2 mg/kg/day or 60 mg/m^2^/day of steroids for less than 4 weeks, and no recurrence was found in outpatient follow-up for more than 3 years. SDNS/FRNS means steroid sensitive, but relapse within 2 weeks after two successive dose reduction or withdrawal. At the same time, it means that the recurrence is more than 2 times in half a year or more than 4 times in 1 year.

This study was registered with the China Clinical Trial Registration Center (registration number: ChiCTR1900023187) and approved by the Ethics Committee of Children's Hospital of Chongqing Medical University. All participants and their guardians understood the content and significance of the study and signed informed consent forms before participating.

### Clinical data

Clinical information, including general information, initial examination/test results, drug response, clinical types, and clinical sub-phenotypes (SSNSWR or SDNS/FRNS) were collected by clinicians. Urinary protein levels, clinical manifestations, and treatment response of patients were monitored during follow-up and evaluated, and disease progression was evaluated over time.

### Genotyping and correlation analysis

Standard procedures were conducted for DNA isolation, genotyping and quality control. Genotyping was carried out at China National Clinical Research Center (Child Health and Disease) using GenCap-WES Capture Kit (MyGenomics, Beijing). HLA alleles were analyzed by four-digit resolution genotyping using xHLA software, which provides fast and accurate HLA typing from short-read next-generation sequencing (NGS) data. Data for 1835 healthy controls were obtained from the database of Beijing Mygenomics Co., Ltd. (the MyGenostics database). We compared the distributions and carrying frequencies of HLA-I/II alleles in children with different clinical sub-phenotypes of SSNS, studied the association of allele carrier status with the clinical characteristics and prognosis of SSNSWR and SDNS/FRNS, and performed univariant/multivariate logistic regression analyses of disease-related indicators to search for risk factors associated with SDNS/FRNS in association with sex and age. We also investigated the relationship between alleles with a frequency > 1% and risk factors for SDNS/FRNS and analyzed underlying interfactor relationships using SDNS/FRNS risk-related HLA alleles to reveal possible mechanisms by which HLA alleles affect the prognosis of children with SSNS.

### Mediating effect analysis

Mediating effect means that the influence relationship between variables is not a direct causal relationship, but is indirectly affected by one or more variables. At this time, indirect variables are called mediating variables, and this effect mechanism is called mediating effect. In our study, mediating effect analysis was used to study the relationship between HLA alleles, C4 and SDNS/FRNS, the predictor variables were HLA allele (HLA-B*15:11, B*44:03 & C*07:06), the mediator variables were C4 level, and the outcome variables were SDNS/FRNS patients.

Four steps of the mediation analysis were involved in the calculation of the mediating effect as follows: Step 1: shows that the predictor variable determines the outcome (Model Y = βTotX) (βTot = total effect); Step 2: shows that the predictor variable affects the mediator (Model M = β1X) (β1 = indirect effect 1); Step 3 shows that the mediator determines the outcome controlling for the predictor (Model Y = β2M + βDirX) (β2 = indirect effect 2, βDir = direct effect); Step 4: in mediation analyses, testing the significance of the mediation effect is equivalent to testing the null hypothesis H0: β2 = 0 versus the alternative hypothesis Ha: β2 ≠ 0.

### Statistical analysis

SAS 9.4 and IBM SPSS 25.0 were employed for statistical analysis. All variables were tested for normality. Measurement data with a normal distribution are expressed as the mean ± standard deviation, and comparisons between groups were conducted with Student’s *t* tests. Measurement data with skewed distributions are described as the median and interquartile range; comparisons between groups were carried out with the Mann–Whitney *U* test. Count data were analyzed by the Chi-square test or Fisher’s exact test, and the results described by case number and rate. Frequencies of HLA alleles were compared between children with different clinical sub-phenotypes of SSNS and healthy controls. A univariate logistic regression model was applied to explore the relationship between HLA alleles and outcomes, and multivariate logistic regression was utilized to explore risk factors for SDNS/FRNS. The results are expressed as odds ratios (OR) or risk ratios plus 95% confidence intervals (95% CIs). Statistical analysis was performed by double-tailed analysis, and the significance level was set as 0.05. A Sobel test was performed to assess the significance of a mediation effect. The false discovery rate (FDR) method was employed to correct the *P* value. All statistical analyses were performed using SAS 9.4 software (Copyright ©2016 SAS Institute Inc. Cary, NC, USA).

## Results

### Differences in clinical characteristics between SSNSWR and SDNS/FRNS

The clinical details of the patients screened are presented in Table 1. A total of 241 patients were enrolled in our study, including 125 SSNSWR and 116 SDNS/FRNS, with a median age of 42 months (range 26–71 months) and a sex ratio (male:female) of 2.8:1, which consistent with published studies [29]. To investigate the differences in clinical characteristics between SSNSWR and SDNS/FRNS, we compared the clinical indicators closely related to the disease. Compared with SSNSWR, SDNS/FRNS exhibited decreased levels of complement 3 (C3) and complement 4 (C4) at onset (1.19 ± 0.23 vs. 1.08 ± 0.25, *P* < 0.001; 0.27 ± 0.12 vs. 0.24 ± 0.11, *P* = 0.018, respectively). Although proteinuria in both groups received initial sufficient steroid treatment was relieved within 4 weeks, the time to first remission after initial steroid treatment in SDNS/FRNS was significantly longer than that in SSNSWR (9.72 ± 4.59 vs. 13.91 ± 9.59, *P* < 0.001).

**Table 1:** Compared with SSNSWR, SDNS/FRNS exhibited lower levels of C3 and C4 at onset and longer time to first remission

Clinical items	Overall	SSNS		*P*
		SSNSWR	SDNS/FRNS	
Number	241	125	116	
Male, *n* (%)	182 (75.5)	104 (83.2)	78 (67.2)	
Age (mon)	42 (26, 71)	42 (27, 69)	41 (25, 72)	0.931
eGFR at onset (mL/min × 1.73 m^2^)	144.76 ± 51.03	144.86 ± 51.02	144.64 ± 51.25	0.973
Cr (µmol/L)	28.9 (22.0, 37.0)	28 (22, 36)	30.25 (23.50, 38.75)	0.150
UA (µmol/L)	310 (245, 365)	304 (242, 361)	318 (251, 391)	0.200
TC (mmol/mL)	9.82 (8.08, 11.67)	10.00 (8.39, 11.76)	9.47 (7.63, 11.47)	0.221
TG (mmol/mL)	2.79 (1.87, 4.09)	2.91 (1.89, 4.03)	2.72 (1.87, 4.11)	0.762
IgG (g/L)	2.67 (1.77, 3.92)	2.54 (1.87, 3.53)	2.85 (1.61, 4.38)	0.601
IgA (g/L)	1.04 (0.72, 1.37)	1.00 (0.68, 1.30)	1.07 (0.76, 1.42)	0.339
IgM (g/L)	1.75 (1.35, 2.38)	1.77 (1.38, 2.22)	1.74 (1.34, 2.47)	0.741
IgE (IU/mL)	313.32 (80.60, 743.15)	311.86 (79.40, 681.41)	324.69 (83.55, 818.65)	0.743
C3 at onset (g/L)	1.14 ± 0.24	1.19 ± 0.23	1.08 ± 0.25	** < 0.001**
C4 at onset (g/L)	0.26 ± 0.11	0.27 ± 0.12	0.24 ± 0.11	**0.018**
Time to first remission (d)	11.74 ± 7.7	9.72 ± 4.59	13.91 ± 9.59	** < 0.001**

The eGFR was calculated by the Schwartz formula: GFR (mL/min × 1.73 m^2^) = 0.413 × Ht/Scr [Scr: 1 mg/dL = 88.4 µmol/L; Ht: height (cm); Scr: serum creatinine (mg/dL)]. Continuous variables with a normal distribution are presented as the mean ± standard deviation; categorical variables or continuous variables with a nonnormal distribution are presented as the median (interquartile range). *P* values lower than 0.01 are shown in bold (SSNSWR vs. SDNS/FRNS). *SSNSWR* steroid-sensitive nephrotic syndrome without recurrence, *SDNS* steroid-dependent nephrotic syndrome, *FRNS* frequently relapse nephrotic syndrome, *eGFR* estimated glomerular filtration rate, *Cr* creatinine, *UA* uric acid, *TC* total cholesterol, *TG* triglyceride, *Ig* immunoglobulin, *C3* complement 3, *C4* complement 4

We further conducted related indicators and univariate logistic regression to analyze factors affecting therapy response and prognosis in SSNS (variables with *P* < 0.05 in the univariate logistic regression analysis were included in multivariate analysis) (Table 2). After correcting for sex, age at onset and other confounding factors, this study suggested that the significant decreased C4 level in children with SDNS/FRNS, which indicate that it may be an independent risk factor for these patients (*P* = 0.008, OR = 0.174, 95% CI 0.048–0.630).

**Table 2:** Complement 4 at onset level indicating that it may be an independent risk factor for SDNS/FRNS

Predictors	*P*	OR	95% CI
Single factor logistic regression			
Age (mon)	0.750	1.001	0.994, 1.009
eGFR at onset (mL/min × 1.73 m^2^)	0.973	1.000	0.995, 1.005
BUN (mmol/mL)	0.089	1.069	0.990, 1.154
Cr (μmol/L)	0.095	1.011	0.998, 1.024
UA (mol/L)	0.064	1.002	1.000, 1.004
TC (mmol/mL)	0.365	0.962	0.885, 1.046
TG (mmol/mL)	0.882	0.991	0.876, 1.120
HDL (mmol/mL)	0.068	1.379	0.977, 1.947
LDL (mmol/mL)	**0.020**	0.893	0.811, 0.982
IgG (g/L)	0.602	1.035	0.910, 1.176
IgA (g/L)	0.503	1.144	0.772, 1.694
IgM (g/L)	0.628	1.085	0.780, 1.511
IgE (IU/mL)	0.325	1.000	1.000, 1.001
C3 at onset (g/L)	**0.001**	0.138	0.045, 0.427
C4 at onset (g/L)	**0.023**	0.048	0.004, 0.659
Multiariable logistic regression			
Age (mon)	0.994	1.000	0.992, 1.008
eGFR at onset (mL/min × 1.73 m^2^)	0.713	0.999	0.992, 1.005
LDL (mmol/mL)	**0.016**	0.883	0.797, 0.977
C4 at onset (g/L)	**0.008**	0.174	0.048, 0.630

Single-factor logistic regression analysis of factors relevant to clinical practice was carried out. Factors with* P* < 0.05 or that were influential in the univariate analysis were included in the multivariate logistic regression analysis to identify independent risk factors for SDNS/FRNS in SSNS. *P* values lower than 0.05 are shown in bold. *SDNS* steroid-dependent nephrotic syndrome, *FRNS* frequently relapse nephrotic syndrome, *eGFR* estimated glomerular filtration rate, *BUN* blood urea nitrogen, *Cr* creatinine, *UA* uric acid, *TC* total cholesterol, *TG* triglyceride, *Ig* immunoglobulin, *HDL* high density lipoprotein, *LDL* low density lipoprotein, *C3* complement 3, *C4* complement 4, *OR* odds ratio, *CI* confidence interval

### HLA-A*11:01 is SSNSWR and SDNS/FRNS-associated alleles specific to Chinese Han children

**Fig. 1:**
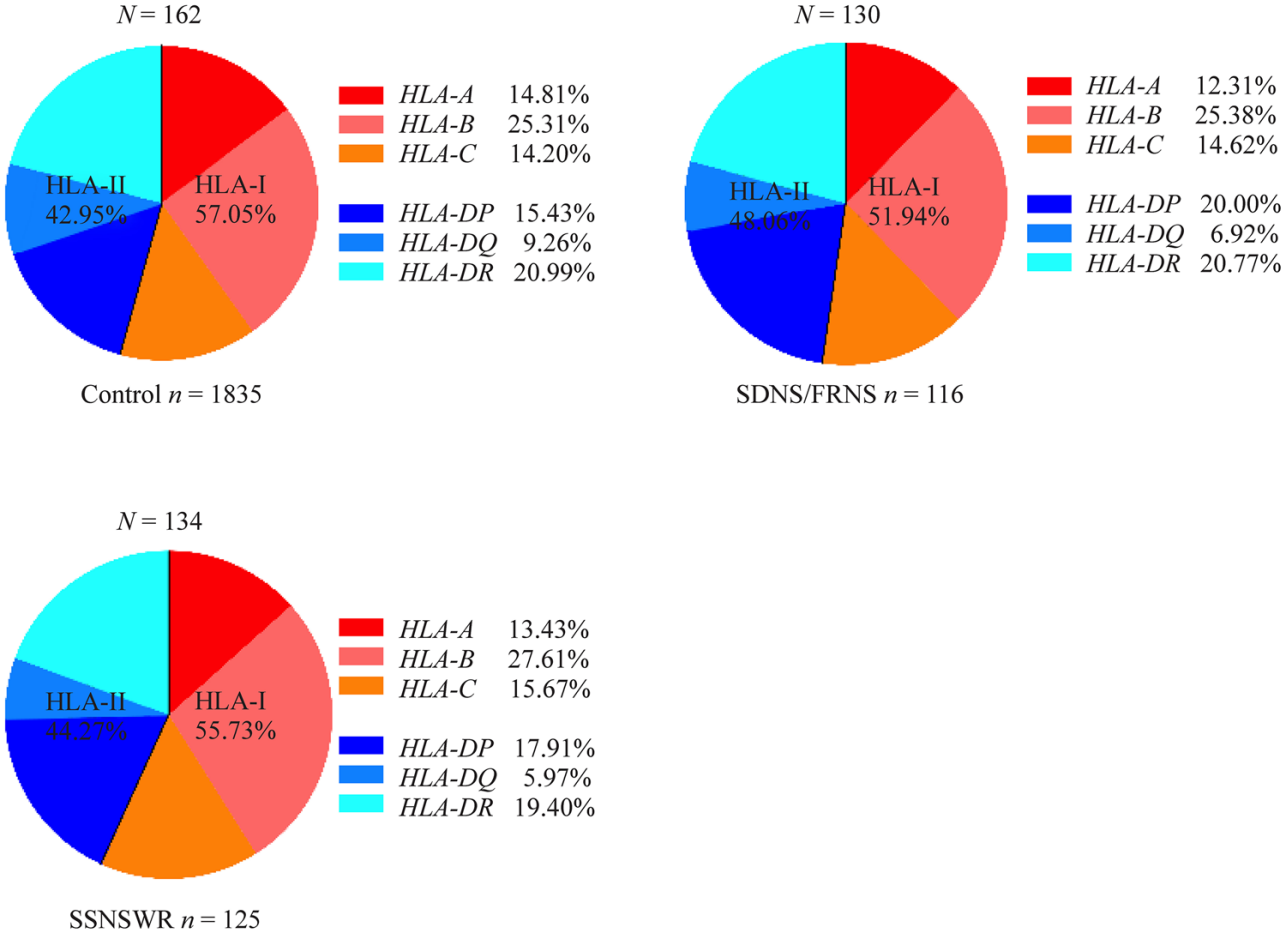
The distribution of HLA-I/II alleles and their six loci were average among the healthy control, SSNSWR and SDNS/FRNS. *HLA* human leukocyte antigen, *SSNSWR* steroid-sensitive nephrotic syndrome without recurrence, *SDNS* steroid-dependent nephrotic syndrome, *FRNS* frequently relapse nephrotic syndrome

**Fig. 2:**
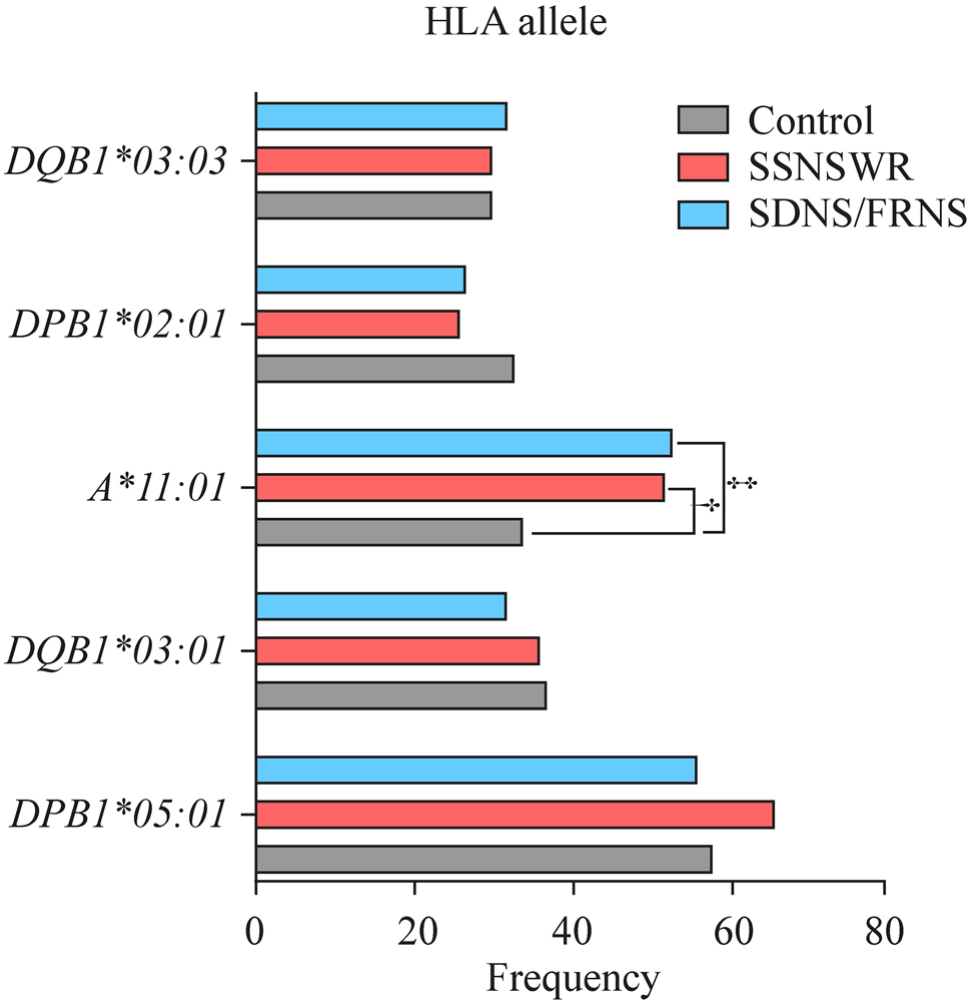
The distributions of the top five HLA alleles with the highest frequencies across healthy control, SSNSWR and SDNS/FRNS and the HLA-A*11:01 allele is highly expressed in SSNSWR and SDNS/FRNS. *HLA* human leukocyte antigen, *SSNSWR* steroid-sensitive nephrotic syndrome without recurrence, *SDNS* steroid-dependent nephrotic syndrome, *FRNS* frequently relapse nephrotic syndrome. ^†^*P* < 0.01, ^‡^*P* < 0.001

To investigate whether HLA alleles influence susceptibility to SSNSWR and SDNS/FRNS, the total patients of the two groups were matched controls, respectively. Interestingly, our study successfully genotyped 178 HLA alleles including 101 class I (*HLA-A*, 27 alleles; *HLA-B*, 48 alleles and *HLA-C*, 27) and 77 class II (*HLA-DPB1*, 27 alleles; *HLA-DQB1*, 16 alleles and *HLA-DRB1*, 34 alleles) alleles. The distributions of the 6 HLA loci among the healthy control, SSNSWR and SDNS/FRNS are provided in Fig. 1. Among these alleles, 27 were expressed only in SSNSWR, the top 5 alleles with the highest frequency at 6 HLA loci were HLA-DPB1*05:01 (66.40%), HLA-A*11:01 (52.00%), HLA-DQB1*03:01 (36.00%), HLA-DQB1*03:03 (30.40%) and B*46:01 (28.00%); 24 were only expressed in SDNS/FRNS and the top 5 alleles with the highest frequency were HLA-DPB1*05:01 (56.03%), HLA-A*11:01 (52.59%), HLA-DQB1*03:01 (31.90%), HLA-DQB1*03:03 (31.90%) and HLA-DRB1*09:01 (30.07%). Compared with the control, the frequency of the HLA-A*11:01 allele was significantly higher in the SSNSWR and SDNS/FRNS (Supplementary Table 2, Fig. 2).

### Risk- and protection-HLA alleles for SSNSWR and SDNS/FRNS

In order to confirm the region-specific susceptibility HLA alleles that associated with steroid dependent and frequent relapse, we further indirectly compared patients’ group (SSNSWR and SDNS/FRNS) with the control. After FDR correction, 6 loci showed statistical significance (DQB1*06:02, A*11:01, B*15:02, DPB1*107:01, A*02:01, A*02:03; *P*-FDR < 0.01). Taken together, these results indicated that HLA-A*11:01, HLA-B*15:02 and HLA-DPB1*107:01 are risk alleles for SSNSWR but that HLA-DQB1*06:02 is a protective allele. In SDNS/FRNS, HLA-A*02:03 and HLA-A*11:01 are risk alleles, but HLA-A*02:01 and HLA-DQB1*06:02 are protective. The details of the difference in HLA allele frequency are listed in Table 3.

**Table 3:** Differences of HLA allele carrier frequencies

HLA allele	Frequency					*P1*	*P1*_FDR	OR1 (95% CI)	*P2*	*P2*_FDR	OR2 (95% CI)
	Control		SSNSWR		SDNS/FRNS						
Protection alleles											
A*01:01	7.68	1.60		0.00		0.0114	0.0835	0.242 (0.068, 0.862)	0.0019	0.0131	0.051 (0.003, 0.839)
A*02:01	25.78	16.80		8.62		0.0253	0.0999	0.592 (0.368, 0.953)	0.0000	**0.0006**	0.284 (0.149, 0.540)
A*30:01	11.39	5.60		3.45		0.0455	0.1365	0.491 (0.231, 1.044)	0.0078	0.0422	0.311 (0.119, 0.807)
DQB1*06:02	16.84	4.00		1.72		0.0002	**0.0024**	0.225 (0.095, 0.534)	0.0000	**0.0003**	0.108 (0.031, 0.380)
Susceptible alleles											
A*02:03	5.07		12.00		12.93	0.0010	0.0137	2.615 (1.474, 4.639)	0.0003	**0.0030**	2.846 (1.600, 5.062)
A*11:01	33.95		52.00		52.59	0.0000	**0.0012**	2.105 (1.465, 3.026)	0.0000	**0.0006**	2.155 (1.480, 3.138)
B*15:02	4.63		15.20		10.34	0.0000	**1.62E−05**	3.749 (2.203, 6.379)	0.0060	0.1451	2.450 (1.307, 4.592)
DQB1*05:02	12.59		21.60		18.10	0.0039	0.0315	1.935 (1.240, 3.021)	0.0859	0.3435	1.560 (0.957, 2.543)
DPB1*107:01	3.16		11.20		6.90	0.0001	**0.0030**	3.951 (2.147, 7.270)	0.0550	0.5351	2.380 (1.124, 5.041)

Differences of HLA allele carrier frequencies between control and SSNSWR; differences of HLA allele carrier frequencies between control and SDNS/FRNS. There was no significant difference in the frequency of HLA alleles between SSNSWR and SDNS/FRNS. The allele carrier frequency is expressed as a percentage. *P* values lower than 0.01 are shown in bold (*P1* control vs. SSNSWR, *P2* control vs. SDNS/FRNS). *HLA* human leukocyte antigen, *SSNSWR* steroid-sensitive nephrotic syndrome without recurrence, *SDNS* steroid-dependent nephrotic syndrome, *FRNS* frequently relapse nephrotic syndrome, *FDR* false discovery rate, *CI* confidence interval, *OR* odds ratio

**Fig. 3:**
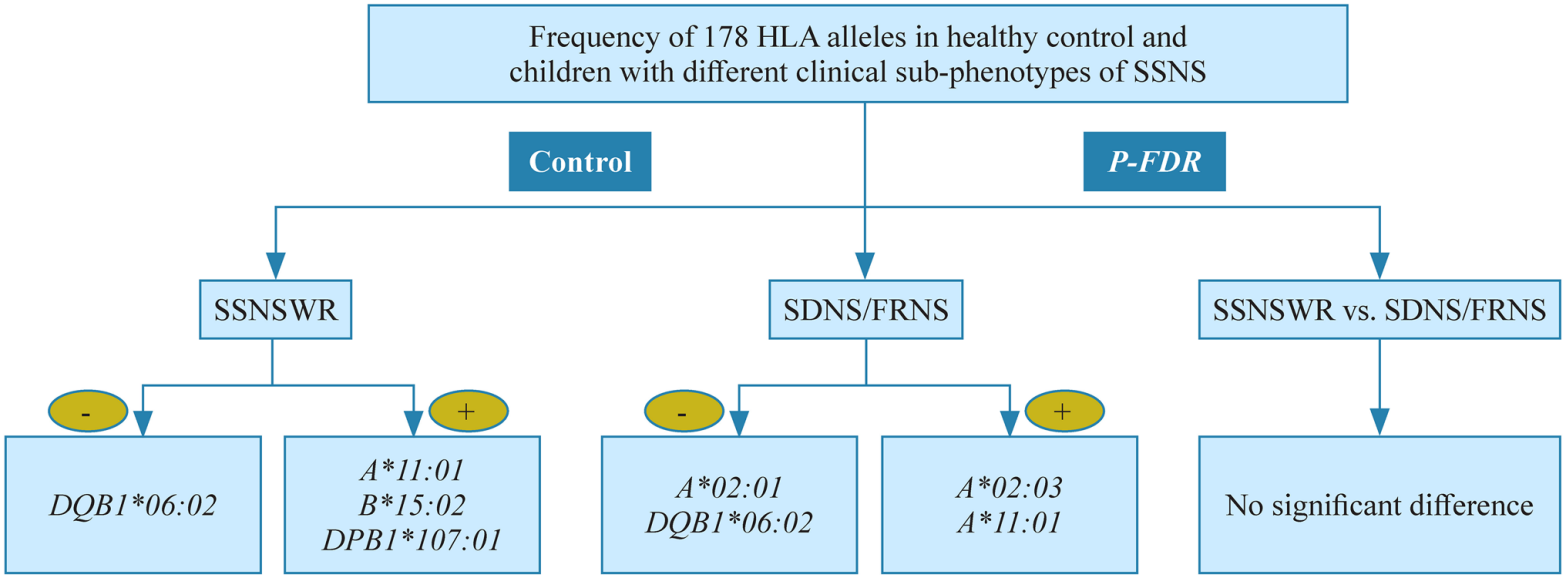
Differences in HLA allele frequencies between children with different clinical sub-phenotypes of SSNS. HLA-A*11:01, HLA-B*15:02 and HLA-DPB1*107:01 were risk alleles for SSNSWR, while HLA-DQB1*06:02 was a protective allele. For SDNS/FRNS, HLA-A*02:03 and HLA-A*11:01 were risk alleles, while HLA-A*02:01 and HLA-DQB1*06:02 were protective alleles. *HLA* human leukocyte antigen, *SSNSWR* steroid-sensitive nephrotic syndrome without recurrence, *SDNS* steroid-dependent nephrotic syndrome, *FRNS* frequently relapse nephrotic syndrome. + risk alleles, − protective alleles

Furthermore, we also found that compared with the control, both SSNSWR and SDNS/FRNS exhibited differential frequency of the alleles HLA-A*11:01 and HLA-DQB1*06:02, with high-frequency of HLA-A*11:01 and low-frequency of HLA-DQB1*06:02 in SSNSWR and SDNS/FRNS (Fig. 3). These findings suggest that HLA-A*11:01 and HLA-DQB1*06:02 are potential risk/protective alleles. Nevertheless, no FDR-adjusted significance was observed between SSNSWR and SDNS/FRNS.

### HLA alleles influence the clinical features of SSNSWR and SDNS/FRNS

To explain the relationship between HLA system and patients’ group (SSNSWR and SDNS/FRNS), we have further analyzed the effect of different alleles on clinical characteristics in these two groups. The results showed that the carrier status of HLA-I and HLA-II alleles affects the onset age, immune status, complement levels and the time to first remission in each group and the HLA-I (*HLA-A*, *HLA-B*, and *HLA-C*) alleles were the most common among the two groups (Table 4).

**Table 4:** Correlation between HLA different alleles and clinical characteristics of SSNSWR and SDNS/FRNS

HLA allele	Clinical items	Overall	Positive	Negative	*P*
HLA different alleles and clinical characteristics of SSNSWR					
B*15:02	Age (mon)	42 (27, 69)	29 (20, 48)	43 (29, 72)	0.042
	C3 at onset	1.19 ± 0.23	1.29 ± 0.24	1.17 ± 0.22	0.046
	C4 at onset	0.26 (0.20, 0.31)	0.29 (0.24, 0.34)	0.25 (0.19, 0.30)	0.036
DQB1*06:02	Age (mon)	42 (27, 69)	74 (72, 124)	42 (27, 61)	0.044
DPB1*107:01	LDL (mmol/mL)	6.37 (4.48, 8.10)	7.70 (6.56, 9.15)	6.06 (4.32, 7.93)	0.022
	C3 at onset	1.19 ± 0.23	1.34 ± 0.21	1.17 ± 0.22	0.008
HLA different alleles and clinical characteristics of SSNSWR and SDNS/FRNS					
A*02:01	UP(-) (d)	13.91 ± 9.59	11.1 ± 3.07	14.17 ± 9.95	0.032
A*02:03	TC	9.47 (7.63, 11.47)	10.42 (9.48, 13.96)	9.42 (7.41, 11.06)	0.040
A*11:01	Age (mon)	41 (25, 72)	46 (31, 77)	33 (21, 62)	0.039

*HLA* human leukocyte antigen, *SSNSWR* steroid-sensitive nephrotic syndrome without recurrence, *SDNS* steroid-dependent nephrotic syndrome, *FRNS* frequently relapse nephrotic syndrome, *LDL* low density lipoprotein, *C3* complement 3, *C4* complement 4, *TC* total cholesterol*, UP(-)* the time to first remission

### HLA-B*15:11, HLA-B*44:03 and HLA-C*07:06 act an intermediary role in SDNS/FRNS

Although several HLA alleles were differentially observed in patient’s group (SSNSWR and SDNS/FRNS) compared with healthy control, there was no FDR-adjusted significance was detected between SSNSWR and SDNS/FRNS. These results suggested an indirect regulation/modification mechanism by which HLA alleles affect the development of SSNS in children with different clinical sub-phenotypes. In order to explore this possible mechanism, we conducted a mediating effect analysis. Based on logistic regression analysis model, HLA-B*15:11, HLA-B*44:03 and HLA-C*07:06 correlated significantly with the C4 level in SDNS/FRNS (Table 5). Interestingly, this result is consistent with above result that C4 is an independent risk factor for SDNS/FRNS.

**Table 5:** Effects of HLA alleles on SDNS/FRNS risk in SSNS

HLA allele	C4			Total indirect effect
	*P*	*P*_FDR	OR	
B*15:11	**0.0006**	0.0112	643.879	− 0.058
B*44:03	**0.0010**	0.0112	331.997	− 0.054
C*07:06	**0.0007**	0.0100	545.960	− 0.066

HLA-B*15:11, HLA-B*44:03 and HLA-C*07:06 were correlated with the C4 level at onset. Included these three alleles in the mediating effect analysis, and a correlation between the risk factors and SDNS/FRNS in SSNS patients was found. Indirect effect 1: β1, between HLA allele and C4; indirect effect 2: β2, between C4 and SDNS/FRNS; total indirect effect: βInd = β1 × β2. Logistic regression analysis was used to explore the relationship between alleles with frequencies > 1% (frequency = 3%) and risk factors for SDNS/FRNS, and the *P* value was corrected by the FDR method. *P* values lower than 0.05 are shown in bold. *HLA* human leukocyte antigen, *SDNS* steroid-dependent nephrotic syndrome, *FRNS* frequently relapse nephrotic syndrome, *SSNS* steroid-sensitive nephrotic syndrome, *C4* complement 4, *FDR* false discovery rate, *OR* odds ratio

## Discussion

Previous studies have shown that HLA allele polymorphism is closely related to the susceptibility, development and prognosis of SSNS. At present, the research on the correlation between HLA and SSNS at home and abroad mainly focuses on the prediction of high-risk genes. Since *HLA-DR7* was first reported as the risk locus of SSNS in the 1980s, the correlation between *HLA-DQA1, DQB1, DR/DQ, DRB1* and SSNS have been found and verified, indicating that HLA alleles play an important role in the occurrence and development of SSNS (Supplementary Tables 3, 4).

However, previous studies also showed that children with SSNS of different races and regions have genetic heterogeneity in disease susceptibility, treatment effect and prognosis: there is an association between *HLA-DR7* antigen and SSNS in Caucasian, Chinese and Arab patients, and the frequency of *HLA-DQB1:0302* in Japanese population is increased; Bouissou et al. found that the presence of *HLA-DR7, DR3/7* or *DQ2* suggests that patients with SSNS are very likely to have frequent recurrence/hormone dependence in the course of the disease. In German children with frequent recurrence, carrying *HLA-DR7* suggests that the treatment response to cyclophosphamide or amphetamine is worse, while Indian children with SSNS have higher expression frequency of *HLA-BW44*, which may affect the efficacy of cyclophosphamide [15, 18–22, 30]. Taken together, it suggests an importance of distinguishing race and region in HLA allele research.

HLA gene complex, one of the most polymorphic regions of the genome, has been reported as the most polymorphic genetic system in humans. By analyzing the genetic and clinical characteristics in SSNSWR and SDNS/FRNS, we found that HLA alleles polymorphisms may affect susceptibilities to different clinical sub-phenotypes of SSNS. HLA-A*11:01 may confers susceptibilities to SSNSWR and SDNS/FRNS while HLA-DQB1*06:02 plays a protective role. A lower level of C4 from logistic regression analysis indicated a higher likelihood of steroid dependence and/or recurrence in a long term. Additionally, HLA-I alleles (HLA-B*15:11, HLA-B*44:03, and HLA-C*07:06) are positively correlated with C4 level. Further mediating effect analysis suggested that HLA-B*15:11, HLA-B*44:03, and HLA-C*07:06 may participate in the regulation of immune recognition, response and homeostasis by affecting the C4 level, which indirectly affects the progression and long-term prognosis of children with SDNS/FRNS. Therefore, our study highlighted the association of the HLA alleles with SSNSWR and SDNS/FRNS, investigated the potential impact of HLA alleles polymorphisms on clinical characteristics, treatment response and prognosis, which aids in drug screening, clinical classification and individualization of treatment in Chinese Han children.

As one of the most common HLA-I alleles in Asian population, HLA-A*11:01 is a susceptible allele often carried in SRNS children in Singapore and end stage renal disease children in China with a high frequency. Studies on the structure of human alloantibodies bound to HLA-A*11:01 demonstrated its association with a variety of autoimmune diseases, including rheumatoid arthritis and skin diseases. A high frequency of this allele is also reported in cases of infection and parasitic diseases (for example, toxoplasma gondii infection) [31–33]. Together, these results suggest an important role of HLA-A*11 in the development of T cell-mediated abnormal immune responses [34–36], thus HLA-A*11:01 might affect the immune response and participate in the occurrence and development of SSNSWR and SDNS/FRNS by regulating T cell antigen presentation.

In this study, the frequency of HLA-A*11:01 in SSNSWR and SDNS/FRNS was significantly higher than that in the healthy control, which suggested it to be a predicting factor for children who are predisposed toward SSNSWR and SDNS/FRNS. Unlike HLA-A*11:01, HLA-DQB1*06:02 was expressed at significantly lower levels in SSNSWR and SDNS/FRNS than that in the control group. HLA-DQB1*06:02 allele and its high-frequency haplotypes have been previously reported to play different (risk/protection) roles in diseases of endocrine system, nervous system and immune system, thus indicating extensive and significant genetic effect [37–42]. In our study, low frequencies of HLA-DQB1*06:02 in SSNSWR and SDNS/FRNS suggested it to be a protective role in regulating the immune response and affecting the occurrence and development of the disease.

Complement plays an important role in regulating antigen-specific immunity, maintaining immune homeostasis, promoting tissue and vascular regeneration, and avoiding autoimmune-based injury [43–45]. Abnormal activation/inhibition of the complement system has been reported in a variety of autoimmune diseases as well as kidney and vascular diseases [46–49]. C4 is the second molecule activated in the classical complement pathway, and it participates in regulating complement activation, preventing immune complex deposition and neutralizing viruses. C4 (C4A and C4B) genes exist in the MHC genome region, between class I and class II HLA genes. Classical complement proteins help to eliminate fragments of dead and damaged cells, therefore attenuating the visibility of diverse intracellular proteins to the adaptive immune system.

Our results of univariate/multivariate logistic regression analyses proved the C4 level to be an independent risk factor for SDNS/FRNS. Compared with SSNSWR, a lower level of C4 was detected in SDNS/FRNS. In the correlation analysis between HLA alleles and risk factors, the loci with carrying frequency less than 1% (*n* is less than 2) were excluded due to statistical considerations to ensure a certain level of statistical efficiency. Using logistic regression analysis, we found that HLA-B*15:11, B*44:03 & C*07:06 were positively correlated with C4 level, suggesting that there may be a correlation between complement and HLA alleles in SSNS.

In the current study of SSNS, there is no report on the levels of HLA-B*15:11, B*44:03 & C*07:06 and C4, but there are similar findings in other diseases, that is, the relationship between HLA and complement. Studies have shown that the total copy number of C4 gene is associated with the risk of autoimmune diseases such as systemic lupus erythematosus (SLE), and this association is considered to be caused by linkage disequilibrium (LD*) with nearby HLA gene alleles [50, 51]. In the European population, DRB1*03:01 has a strong LD (*R*^2^ = 0.71) with the common C4B-b (s) allele. Many MHC single nucleotide polymorphism associated with SLE and Stevens Johnson syndrome (SJS) have a proportional LD with C4 & DRB1*03:01 [52].

Up to now, there are few reports on these three HLA alleles: HLA-B*15:11 was first verified by Hildebrand in Korean patients, and the allele is most likely evolved from B*1501 point mutation/short fragment exchange/transformation, encoding fragment of B75 antigen [53]. In 2000, a strong correlation between B*44032-CW*0706 (HLA-C*07:06) was found in Bubi population [54]. HLA-B*44:03: serious allergic reaction of skin and mucosa with complex pathogenesis including SJS and toxic epidermal necrolysis (TEN) is considered to be a non-immediate hypersensitivity reaction mediated by T cells. Studies have shown that HLA-B*44:03 is a common marker of SJS/TEN and severe cellular surface complications (SOC) in Eurasian populations such as Europe, India, Japan and Thailand. Haplotype analysis shows that HLA-B*44:03-HLA-C*07:01 is a potential risk factor for CM-SJS/TEN combined SOC in Thai population [55]; In 2020, the research results reported by Wang et al. clarified the expression profile and expression degree of several complement components in niDHRs, especially SJS and TEN, at the mRNA and protein levels for the first time. To a certain extent, it indicates that the abnormality of complement system may be a part of the pathogenesis of SJS and TEN [56]. Combined with the results showing the impact of HLA alleles on the complement system of other diseases, we speculate that there may be LD between HLA-B*15:11, B*44:03 & C*07:06 and C4 or C4-related complement genes. These three alleles may affect C4 gene expression or copy number via this potential association, and it in turn affects C4 levels, participating in immune recognition, response and homeostasis, and indirectly affecting treatment response and prognosis. In addition, our results suggested a higher likelihood of steroid dependence/relapse all over the course of the disease.

In conclusion, the HLA-A*11:01 allele is associated with SSNSWR and SDNS/FRNS in Chinese Han children, which emphasizes the value of HLA alleles’ detection in precise typing of Chinese children with SSNS. HLA-B*15:11, HLA-B*44:03 and HLA-C*07:06 may indirectly affect the prognosis of children with SDNS/FRNS by regulating C4 level. Our research aims to provide a new direction for pathogenesis, functional studies and individualized clinical diagnosis and treatment strategies for SSNS patients. Also, this research provided an important reference for HLA-I allele (including HLA-A*11:01, HLA-B*15:11, HLA-B*44:03 and HLA-C*07:06) in further genetic counseling and testing for inherited gene mutations in SSNSWR or SDNS/FRNS.

## Supplementary Information

Below is the link to the electronic supplementary material.

Supplementary file1 (DOCX 22 kb)

## Data Availability

The data used to support the findings of this study are available from the corresponding author upon request.
