# Supplementary material for: Association between HLA alleles and sub-phenotype of childhood steroid-sensitive nephrotic syndrome
Source: World J Pediatr. 2022 Jan 1;18(2):109–19. doi: 10.1007/s12519-021-00489-y (PMC8843916; doi:10.1007/s12519-021-00489-y)
Supplement: Supplementary file 1 — Supplementary file1 (DOCX 22 kb) [file 12519_2021_489_MOESM1_ESM.docx]

**Supplementary Table 1.** Inclusion and exclusion criteria for our study

| **Inclusion** | **Exclusion** |
| --- | --- |
| Onset age: 1-18 y | Steroid side effects were obvious, intolerable or contraindications |
| Followed up: more than 2 y | Missing of first medical records/lost of follow-up |
| Idiopathic nephrotic syndrome diagnostic criteria^a^： | Secondary nephrotic syndrome: |
| Large amount of proteinuria: 24 h proteinuria ≥ 50 mg/kg or morning urine protein/creatinine (mg/mg) ≥ 2.0, 3 times a wk morning urine protein qualitative (+++-++++) | Includes purpura nephritis, lupus nephritis, hepatitis B virus associated **glomerulonephritis,** antineutrophil cytoplasmic antibody related nephritis, etc |
| Hypoproteinemia: serum albumin is lower than 25 g/L | Congenital nephrotic syndrome |
| Hyperlipidemia: serum cholesterol is higher than 5.7 mmol/L |  |
| Different degrees of edema |  |

^a^The first two criteria were the necessary conditions for diagnosis

**Supplementary Table 2.** The distributions of the HLA alleles with the top ten frequencies between the control, SSNSWR and SDNS/FRNS

| **Control (*n* = 1835)** | | **SSNSWR (*n* = 125)** | | **SDNS/FRNS (*n* = 116)** | |
| --- | --- | --- | --- | --- | --- |
| **HLA allele** | **%** | **HLA allele** | **%** | **HLA allele** | **%** |
| *DPB1*05:01* | 58.15 | *DPB1*05:01* | 66.40 | *DPB1*05:01* | 56.03 |
| *DQB1*03:01* | 37.33 | *A*11:01* | 52.00 | *A*11:01* | 52.59 |
| *A*11:01* | 33.95 | *DQB1*03:01* | 36.00 | *DQB1*03:01* | 31.90 |
| *DPB1*02:01* | 33.41 | *DQB1*03:03* | 30.40 | *DQB1*03:03* | 31.90 |
| *DQB1*03:03* | 30.25 | *DRB1*09:01* | 28.00 | *DRB1*09:01* | 30.17 |
| *DRB1*09:01* | 27.90 | *B*46:01* | 28.00 | *C*07:02* | 28.45 |
| *C*01:02* | 27.30 | *C*07:02* | 26.40 | *DPB1*02:01* | 26.72 |
| *A*24:02* | 26.59 | *B*40:01* | 26.40 | *C*01:02* | 26.72 |
| *C*07:02* | 26.38 | *DPB1*02:01* | 25.60 | *B*40:01* | 24.14 |
| *A*02:01* | 25.78 | *A*02:07* | 24.80 | *A*24:02* 21.55 | |

*HLA* human leukocyte antigen, *SSNSWR* steroid-sensitive nephrotic syndrome without recurrence, *SDNS* steroid-dependent nephrotic syndrome, *FRNS* frequently relapse nephrotic syndrome

**Supplementary Table 3.** Previous studies of correlation between HLA alleles and SSNS

| **Population** | **Findings (susceptible alleles)** | **Authors** | **Year** |
| --- | --- | --- | --- |
| France (*n* = 54) | *DR7* | De Mouzon et al. | 1981 |
| Caucasoid (*n* = 40) | *DR7, DQw2* | Godfrey et al. | 1990 |
| Japanese (*n* = 36) | *DR7, DQw2* | Jin et al. | 1991 |
| Southwest France (*n* = 65) | *DR7, DQB1:0201, DQA1*0201,*0301, *0302* | Konrad et al. | 1994 |
| Southwest Germany (*n* = 42) | *DR7, DRB1:0301, DQB1:0201* |  |  |
| Chinese Han (*n* = 40) | *DR7* | Zhou et al. | 1994 |
| Japanese (*n* = 24) | *DQB1*0302* | Kasumi Kuramitsu Abe et al. | 1995 |
| Southwest France & Southwest Germany (*n* = 242) | *DR7, DQ2, DR3/DR7* | Bouissou et al. | 1995 |
| Japanese (*n* = 30) | *DQB1*0301, DQB1*0601* | Kobayashi et al. | 1995 |
| Japanese (*n* = 24) | *DQB1*0302* | Kasumi Kuramitsu Abe et al. | 1995 |
| French and a German (*n* = 129) | *DQA1*0201, DQB1*0201* | Haeffner et al. | 1997 |
| Egyptian (*n* = 27) | *DQB1*0601, DRB1*01* | Bakr et al. | 1998 |
| Kuwaiti (*n* = 61) | *DRB 1*0701* | Al-Eisa et al. | 2000 |
| Turkey (*n* = 47) | *B13, Cw5, Cw7, DR4, DR7, DRw10, Drw15(2), DQ2* | Karabay-Bayazit et al. | 2001 |
| Polish (*n* = 127) | *HLA-DR3, DR7, DR3/7, DQ2* | Krasowska-Kwiecień et al. | 2006 |
| Indian (*n* = 100) | *DQ-β1*020X, DR-β1*070X-DQ-β1*020X* | Gulati et al. | 2007 |
| Egyptian (*n* = 37) | *B27, A11, B13* | Donia et al. | 2008 |
| Taiwan of China (*n* = 59) | *DR11* | Huang et al. | 2009 |
| South Asian (*n* = 214) | *DQA1, DQB1* | Gbadegesin et al. | 2015 |
| Indian (*n* = 76) | *DQB1*02* | Ramanathan et al. | 2016 |
| Japanese (*n* = 224) | *DRB1*08:02, DQB1*06:04, DRB1*08:02-DQB1*03:02* | Jia et al. | 2018 |
| African American (*n* = 65) | *HLA-DQA1* | Adeyemo et al. | 2018 |
| Chinese (*n* = 501) | *HLA-DQA1* | Zhu et al. | 2019 |
| European (*n* = 422) | *HLA-DR/DQ* | Dufek et al. | 2019 |

*HLA* human leukocyte antigen, *SSNS* steroid-sensitive nephrotic syndrome

**Supplementary Table 4.** Previous studies of correlation between HLA alleles and SSNS

| **Population** | **Findings (protection alleles)** | **Authors** | **Year** |
| --- | --- | --- | --- |
| Chinese Han (*n* = 40) | *DQ1* | Zhou et al. | 1994 |
| Japanese (*n* = 24) | *DQA1*0103* | Kasumi Kuramitsu Abe et al. | 1995 |
| Southwest France & Southwest Germany (*n* = 242) | *DR2* | Bouissou et al. | 1995 |
|  | *DR6* |  |  |
|  | *DQ1* |  |  |
| Japanese (*n* = 30) | *DQB1*0501* | Kobayashi et al. | 1995 |
| Japanese (*n* = 24) | *DQA1*0103* | Kasumi Kuramitsu Abe et al. | 1995 |
| French and a German (*n* = 129) | *DQA1*0102* | Haeffner et al. | 1997 |
|  | *DQB1*0602* |  |  |
| Egyptian (*n* = 27) | *DRB1*11* | Bakr et al. | 1998 |
| Indian (*n* = 100) | *DR-β1*1001, DR-β1*130X, DQ-β1*030X* | Gulati et al. | 2007 |
| Taiwan of China (*n* = 59) | *DR14* | Huang et al. | 2009 |

*HLA* human leukocyte antigen, *SSNS* steroid-sensitive nephrotic syndrome
